# Morphological and Physio-Chemical Responses to PEG-Induced Water Stress in *Vanilla planifolia* and *V. pompona* Hybrids

**DOI:** 10.3390/ijms24054690

**Published:** 2023-02-28

**Authors:** José Martín Barreda-Castillo, Juan L. Monribot-Villanueva, Noé Velázquez-Rosas, Paul Bayman, José A. Guerrero-Analco, Rebeca Alicia Menchaca-García

**Affiliations:** 1 Centro de Investigaciones Tropicales, Universidad Veracruzana, Xalapa 91090, Veracruz, Mexico; 2 Red de Estudios Moleculares Avanzados, Instituto de Ecología A.C., Clúster Científico y Tecnológico BioMimic®, Xalapa 91073, Veracruz, Mexico; 3 Department of Biology, University of Puerto Rico, Río Piedras, San Juan 00925, Puerto Rico

**Keywords:** climate change, crops, genetic improvement

## Abstract

*Vanilla planifolia* is an orchid of cultural and economic value. However, its cultivation in many tropical countries is threatened by water stress. In contrast, *V. pompona* is a species that is tolerant of prolonged periods of drought. Due to the need for plants’ resistant to water stress, the use of hybrids of these two species is considered. Therefore, the objective of this study was to evaluate the morphological and physio-chemical responses of in vitro vanilla seedlings of the parental genotype *V. planifolia*, and the hybrids *V. planifolia* × *V. pompona* and *V. pompona* × *V. planifolia,* which were then exposed over five weeks to polyethylene glycol-induced water stress (−0.49 mPa). Stem and root length, relative growth rate, number of leaves and roots, stomatal conductance, specific leaf area, and leaf water content were determined. Metabolites potentially associated with the response to water stress were identified in leaves, through untargeted and targeted metabolomics. Both hybrids exhibited a smaller decrease in the morphophysiological responses compared to *V. planifolia* and exhibited an enrichment of metabolites such as carbohydrates, amino acids, purines, phenols, and organic acids. Hybrids of these two species are considered as a potential alternative to the traditional cultivation of vanilla to face drought in a global warming scenario.

## 1. Introduction

Vanilla (*Vanilla planifolia* Andrews) is an important crop worldwide [1]. Its fruit, whose commercial value recently reached USD 600 per kg, is used in food, perfumes, and pharmaceuticals [1,2]. Vanilla’s fruit is appreciated because of its aromatic quality, which is provided by phenolic compounds, of which vanillin is the most important [2]. Despite its economic importance, in Mexico, *V. planifolia* is subject to special protection (NOM-059-SEMARNAT-2019) and it is also protected worldwide by the Convention on International Trade in Endangered Species of Wild Flora and Fauna, and the International Union for Conservation of Nature Red List [3]. *V. planifolia* is seriously threatened due to overexploitation and habitat loss [4]. In addition, genetic erosion has occurred due to the fact that plants for cultivation are always propagated asexually [4,5], making vanilla more susceptible to several biotic and abiotic factors, including water deficiency [5].

Vanilla, like many other crops, is threatened by water stress due to drought, which is expected to increase in the face of climate change [6]. Originally cultivated in Mexico, vanilla is mainly cultivated in tropical countries such as Madagascar and Indonesia [3,4]. Tropical countries will be seriously affected by climate change, with increased temperatures and longer drought periods [6,7]. In a future scenario of climate change, *V. planifolia* will be affected in terms of its growth and development [2,4,8], because it lacks the efficient response mechanisms to deal with water stress [3,4,5]. Therefore, vanilla plants with improved tolerance to water stress are needed.

Plants that are resistant to water stress are capable of adapting their physiology, growth, and anatomy in order to cope with the lack of available water [9]. Roots production and elongation must be increased [10], and stomata are closed in order to reduce the water lost by transpiration [11,12]. Stomatal closure also reduces CO_2_ uptake and consequently decreases photosynthetic activity, which reduces the specific leaf area (SLA) and therefore, the relative growth rate (RGR) [11,13,14]. For this reason, water-stress-tolerant plants usually are smaller in surface area than drought-sensitive plants under full hydration [14].

In addition to the anatomical and physiological responses, plants must modify their metabolism in order to tolerate water stress [11,14]. Due to the decrease in turgor, plants accumulate within their cells’ metabolites, which act as osmolytes to keep membranes and proteins stable [15,16]. Due to the effects of water stress on photosynthesis, reactive oxygen species (ROS) accumulate within the cells causing cell damage [17]. For this reason, the accumulation of metabolites with antioxidant activity is also necessary [17,18]. Consequently, the accumulation of osmolytes and antioxidants, such as carbohydrates [16,19], amino acids [15,20,21], purines [15,20,22], organic acids [23], and phenols [24,25], among other specialized metabolites [26,27], has been reported in plants that are tolerant to water stress.

Through genetic improvement it is possible to obtain plants with enhanced morphological and physio-chemical responses to water stress [28,29]. The generation of hybrids might allow a combination of phenotypical characteristics of the parental species and also the appearance of new characters [28,29,30]. In the case of vanilla, hybrids between local Mexican species *V. planifolia* (PL) (favored for aroma and flavor) [1] and *V. pompona* (PO) (described as more resistant to water stress) [31] could display the necessary tolerance to water stress, while preserving the aromatic qualities required by the industry [4,28,29]. These hybrids have already been shown to have improved resistance to *Fusarium oxysporum* f. sp. *vanillae* [32], vanilla’s most important pathogen*,* but their response to water stress has not been addressed yet.

For the study of water stress, previous research has used osmotically active substances such as polyethylene glycol (PEG) to reduce the water potential in the medium in vitro [33,34]. Few studies have simultaneously focused on the study of morphological and physio-chemical responses to understand the possible mechanisms of tolerance to induced water stress [15,16,35,36,37]. Thus, the objective of this work was to determine the morphological and physio-chemical responses to PEG-induced water stress of *V. planifolia* (PL) and *V. pompona* (PO) hybrids as a potential alternative to face the challenges of climate change.

## 2. Results

### 2.1. Morphological and Physiological Responses to Water Stress

**Figure 1:**
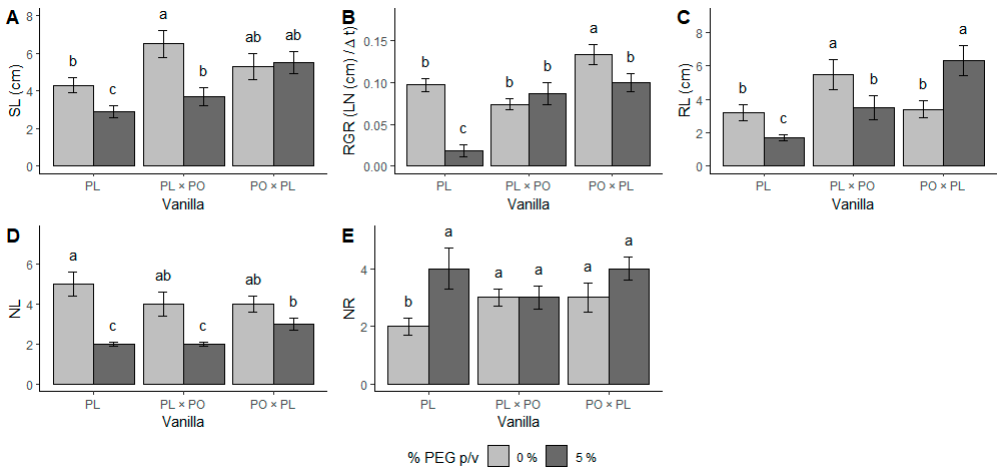
Water stress affects growth of *Vanilla planifolia* (PL) seedlings and *V. planifolia* × *V. pompona* (PL × PO) and *V. pompona* × *V. planifolia* (PO × PL) hybrids. (**A**) Stem length (SL); (**B**) relative growth rate (RGR); (**C**) root length (RL); (**D**) number of new leaves (NL); and (**E**) number of new roots (NR). Gray bars: 0% PEG (control group, −0.08 mPa). Black bars: 5% PEG and hydric stress (−0.49 mPa). Bars with different letters are significantly different (*p* < 0.05), according to GLM test.

The morphological and physiological parameters evaluated in the parental genotype PL, and in the hybrids *V. planifolia* × *V. pompona* (PL × PO) and *V. pompona* × *V. planifolia* (PO × PL) exposed to PEG-induced water stress (−0.49 mPa) during five weeks were stem and root length, relative growth rate (RGR), number of leaves and roots, stomatal conductance (SC), specific leaf area (SLA), and leaf water content (LWC). Stem length (SL) was reduced due to water stress in PL and PL × PO (χ^2^ _(2,57)_ = 8.78, *p* < 0.05), whereas no reduction was observed in PO × PL (Figure 1A). RGR decreased due to water stress in PL and PL × PO (F _(2,57)_ = 6.37, *p* < 0.01). The most affected genotype was PL, while PL × PO RGR was not affected (Figure 1B). The root length (RL) was modified due to water stress in all genotypes evaluated; it decreased in PL and PL × PO and increased in PO × PL (χ^2^ _(2,57)_ = 19.76, *p* < 0.01) (Figure 1C).

**Figure 2:**
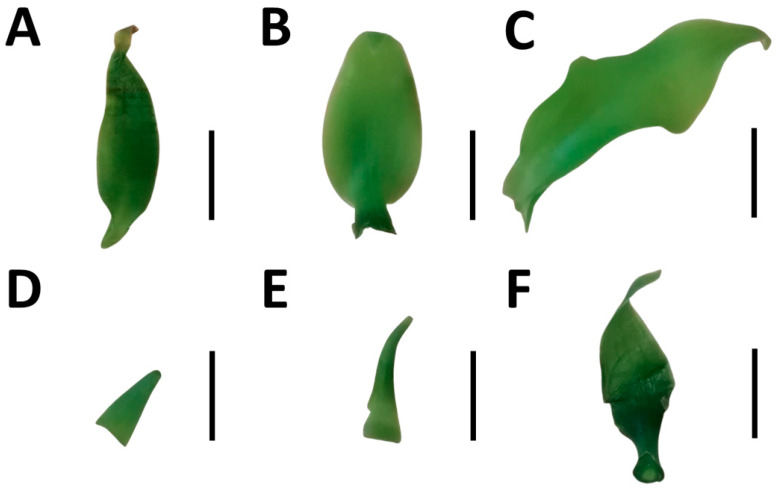
Leaves of vanilla seedlings without and with PEG-induced water stress. (**A**,**D**) *Vanilla planifolia* (PL); (**B**,**E**) *V. planifolia* × *V. pompona* (PL × PO); (**C**,**F**) *V. pompona* × *V. planifolia* (PO × PL); (**A**–**C**) control leaves (−0.08 mPa); and (**D**–**F**) leaves produced under water stress (−0.49 mPa). Scale bars = 10 mm.

The number of new leaves (NL) was significantly reduced by water stress in PL and PL × PO (χ^2^ _(2,57)_ = 6.35, *p* < 0.01) while in PO × PL, it remained unchanged (Figure 1D). In contrast, there was a higher number of new roots (NR) that were produced under water stress in PL (χ^2^ _(2,57)_ = 0.015, *p* < 0.01), which doubled the number of roots in comparison to its controls. However, in the hybrids, differences between the two conditions were not significant (Figure 1E). The leaves of all vanilla genotypes were smaller under water stress (Figure 2D–F) than the controls (Figure 2A–C).

**Figure 3:**
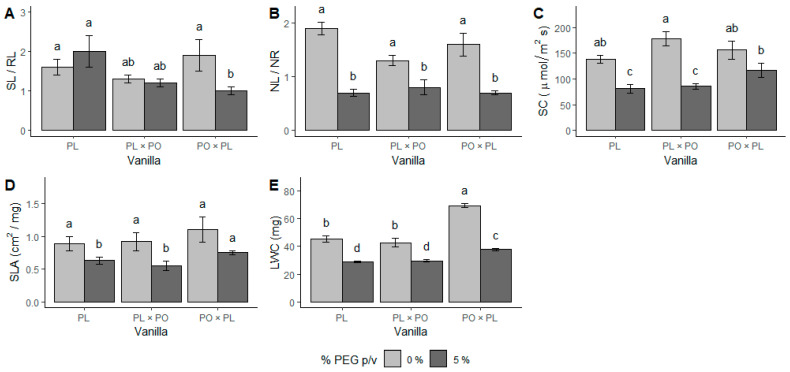
Water stress affects growth ratios and physiology of *Vanilla planifolia* (PL) seedlings and *V. planifolia* × *V. pompona* (PL × PO) and *V. pompona* × *V. planifolia* (PO × PL) hybrids. (**A**) Stem length/longest root length ratio (SL/RL); (**B**) number of leaves/number of roots ratio (NL/NR); (**C**) stomatal conductance (SC); (**D**) specific leaf area (SLA); and (**E**) leaf water content (LWC). Gray bars: 0% PEG (control group, −0.08 mPa). Black bars: 5% PEG, hydric stress (−0.49 mPa). Bars with different letters are significantly different (*p* < 0.05), according to GLM or ANOVA and post hoc Tukey test.

The ratio of stem length/longest root length (SL/RL) was modified by water stress only in the PO × PL hybrid (χ^2^ _(2,57)_ = 16.77, *p* < 0.01), suggesting a sign of the prioritization of roots compared to the leaves (Figure 3A). The ratio of the number of leaves/number of roots (NL/NR) was significantly reduced by water stress in all the vanillas studied (χ^2^ _(2,57)_ = 3.88, *p* < 0.05) (Figure 3B). Stomatal conductivity (SC) decreased due to water stress in PL and in PL × PO (F _(2,57)_ = 41.1, *p* < 0.01). In contrast, the reduction in SC in PO × PL by water stress was not significant (Figure 3C). Similarly, the specific leaf area (SLA) was significantly reduced under water stress in PL and PL × PO, but not in PO × PL (F _(2,57)_ = 12.28, *p* < 0.01) (Figure 3D). All vanillas exhibited a significant decrease in leaf water content (LWC) in water stress (F _(2,57)_ = 14.7, *p* < 0.01) (Figure 3E).

### 2.2. Physio-Chemical Response to Water Stress

**Figure 4:**
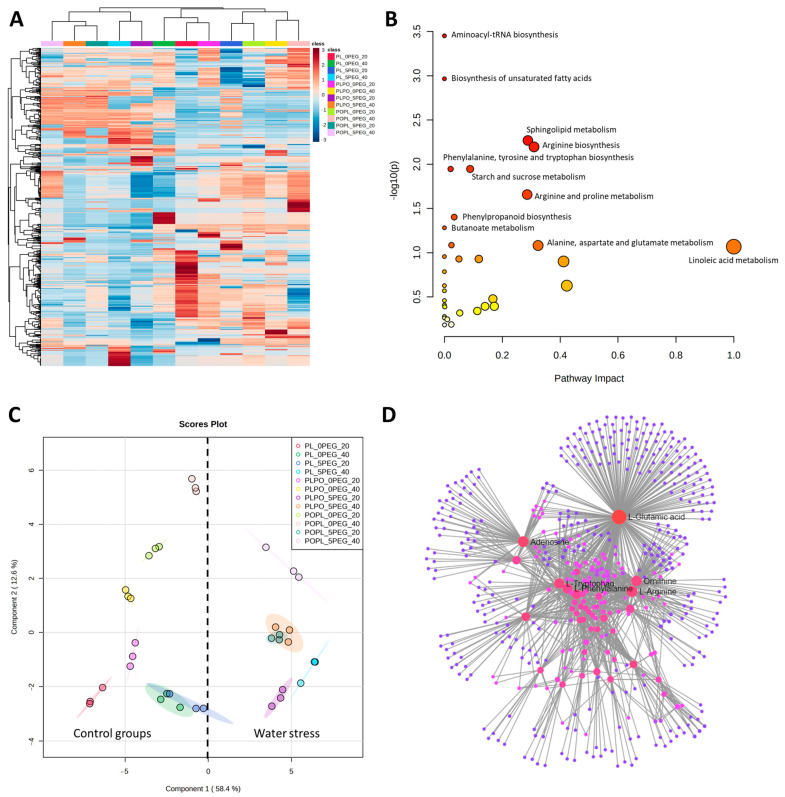
Metabolomics of *Vanilla* plants with and without water stress. (**A**) Hierarchical ordering heatmap, (**B**) pathway analysis, (**C**) principal component analysis (PCA), and (**D**) network analysis of metabolomic data obtained by UPLC-MS-QTOF from vanilla seedlings exposed to 20 and 40 days of PEG-induced water stress. The heatmap was generated using Pearson and Ward for distance measure and clustering algorithm, respectively. The pathway analysis was determined by Mummichog algorithm, KEGG database, and *Oryza sativa* library. PL: *Vanilla planifolia*, PLPO: *V. planifolia* × *V. pompona*, POPL: *V. pompona* × *V. planifolia*, 0 PEG: control group (−0.08 mPa), 5 PEG: hydric stress (−0.49 mPa), 20: 20 days of water stress exposure, and 40: 40 days of water stress exposure.

Untargeted metabolomic analysis based on mass spectrometry detected 812 signals (retention time–mass/charge features; RT-m/z) in the positive mode of ionization (electrospray) and 382 signals in the negative mode. The effect of water stress on the vanilla plants was evidenced by a heatmap (Figure 4A). Two major clusters were identified with distinct patterns of altered metabolite abundances: all the vanillas under full hydration (control groups) were grouped in one cluster, and those exposed to water stress in another, except for PL at 20 days of water stress, which grouped in the clade with all the vanillas under full hydration. Metabolites belonging to the pathways of amino acids, carbohydrates, lipids, phenols, and organic acids, among others, were found (Figure 4B). The names of the identified metabolic pathways, as well as the names of the tentatively identified metabolites, are shown in Tables S1 and S2, respectively. Principal component analysis (PCA) was performed for drought and full hydration plants at the two exposure times. The first component (PC1) explained 58.4% of total variation, whereas the second component (PC2) explained 12.6% variation. The scores between PC1 and PC2 revealed two distinct groups associated with drought and hydration conditions, suggesting a physio-chemical change due to water stress (Figure 4C). Similar to the heatmap (Figure 4A), PL at 20 days of water stress exposure also grouped with all the vanillas under full hydration. The metabolite interaction network analysis (Figure 4D) allowed us to identify the amino acids L-tryptophan, L-phenylalanine, L-arginine, L-glutamic acid, and ornithine, as well the purine adenosine, as main nodes among all the metabolites were detected and tentatively identified.

**Figure 5:**
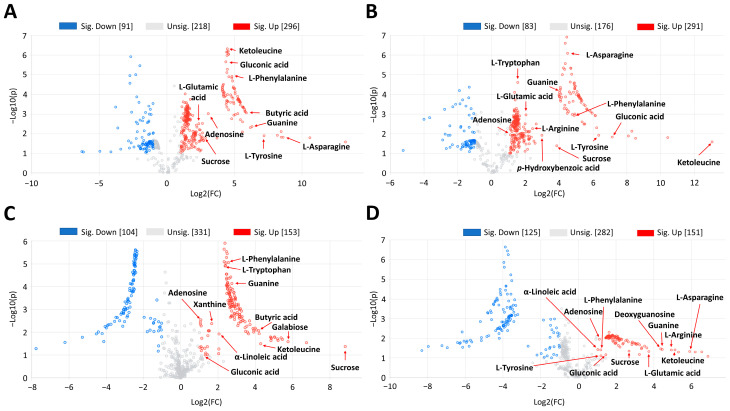
Volcano plots of metabolomic data obtained by UPLC-MS-QTOF comparing *V. planifolia* × *V. pompona* (PL × PO) and *V. pompona* × *V. planifolia* (PO × PL) hybrids versus *V. planifolia* (PL) under water stress exposure. (**A**) PL × PO*/*PL at 20 days of water stress exposure. (**B**) PO × PL*/*PL at 20 days of water stress exposure. (**C**) PL × PO*/*PL at 40 days of water stress exposure. (**D**) PO × PL*/*PL at 40 days of water stress exposure. Some differential metabolites (fold change >2) that are tentatively identified are shown in each subfigure; the complete list is in Tables S3–S6.

In order to go deeper into the physio-chemical changes exhibited by PL, PL × PO, and PO × PL plants, we performed a fold change analysis comparing both hybrids against PL at two different times of water stress exposition (Figure 5). At 20 days of water stress exposure, both hybrids accumulated 9,10-epoxystearic acid, butyric acid, gluconic acid, hexadecanedioic acid, sphinganine, adenosine, guanine, biotin, ketoleucine, L-glutamic acid, L-arginine, L-tryptophan, L-tyrosine, L-phenylalanine, *p*-hydroxybenzoic acid, sucrose, and a disaccharide, in comparison to PL. Xanthine, sphingosine, galabiose, and pheophorbide a were accumulated only in PL × PO; α-linoleic acid, 8,11,14-eicosatrienoic acid, ascorbic acid and L-asparagine were accumulated only in PO × PL (Figure 5A,B; detailed information in Tables S3–S6). At 40 days of water stress exposure, both hybrids accumulated 8,11,14-eicosatrienoic acid, ascorbic acid, butyric acid, gluconic acid, α-linolenic acid, γ-linolenic acid, adenosine, guanine, ketoleucine, L-tryptophan, L-phenylalanine, galabiose, sucrose, and a disaccharide, in comparison to PL. Xanthine was accumulated only in PL × PO, while 9,10-epoxystearic acid, phytosphingosine, sphinganine, sphingosine, deoxyguanosine, L-glutamic acid, L-arginine, L-asparagine, *p*-hydroxybenzoic acid, L-tyrosine, pheophorbide a, and lotaustralin were accumulated only in PO × PL (Figure 5C,D; detailed information in Tables S3–S6).

**Figure 6:**
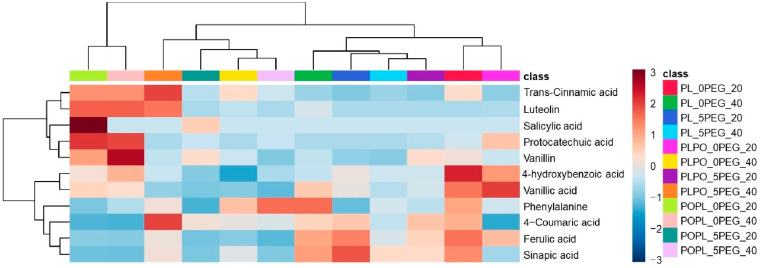
Hierarchical ordering heatmap of phenolic compounds identified by targeted metabolomics as result of exposure of vanilla seedlings to 20 and 40 days of PEG-induced water stress. The heatmap was generated using Pearson and Ward for distance measure and clustering algorithm, respectively. PL: *Vanilla planifolia*, PLPO: *V. planifolia* × *V. pompona*, POPL: *V. pompona* × *V. planifolia*, 0 PEG: hydration status (control group, −0.08 mPa), 5 PEG: hydric stress (−0.49 mPa), 20:20 days of water stress exposure, and 40:40 days of water stress exposure. Complementary information about the phenolic compounds is shown in Table S7.

In addition, ten phenolic compounds and one amino acid were identified and quantified through targeted metabolomics in all the vanillas exposed to water stress and full hydration. The phenolics identified in both conditions were salicylic acid, vanillic acid, vanillin, ferulic acid, sinapic acid, 4-coumaric acid, *trans*-cinnamic acid, luteolin, protocatechuic acid, and 4-hydroxybenzoic acid. In addition, phenylalanine, which is a phenolics precursor, was also identified and quantified (Figure 6; the concentration of each phenolic compound is shown in Table S7). Phenylalanine had the highest concentration among all the compounds quantified. Salicylic acid was detected only in PO × PL at 20 days, and a decrease in its concentration was observed at 40 days. Luteolin was identified in all vanillas under full hydration, but under water stress, it could be observed only in PL × PO at 40 days. The content of vanillic acid, vanillin, ferulic acid, *trans*-cinnamic acid, protocatechuic acid, and 4-hydroxybenzoic acid decreased in all the vanillas, and only sinapic acid and 4-coumaric acid exhibited an enrichment under water stress.

## 3. Discussion

### 3.1. Morphological and Physiological Responses

Plants use various strategies to improve their water management efficiency when water is limited, several of which were observed in the vanilla seedlings exposed to water stress. In this work, we determined the morphological and physio-chemical responses to PEG-induced water stress of *V. planifolia* and *V. pompona* hybrids and the parental *V. planifolia* genotype. PO × PL hybrid increased root length rather than root number, which allowed for an increased water uptake, a strategy observed in water-stress-tolerant orchids [9,38,39], whereas the PL × PO stem length and the number of leaves produced under water stress were reduced, reducing water loss [11,40].

The decrease in LWC is considered a direct indicator of the decrease in water uptake by the plant due to water stress [20]. In order to conserve water, the production of leaves decreases, stomatal closure is induced, and SLA is reduced as well [41,42,43,44,45]. This might explain why PL and PL × PO exhibited the greatest reduction in leaves produced, stomatal closure, and SLA as a possible strategy to conserve water [18]. In contrast, PO × PL exhibited the lowest reduction in LWC, perhaps explaining why it produced more leaves than the other genotypes, and why it was able to maintain the stomata open and not reduce SLA.

Although transpiration is reduced by the morphological responses previously mentioned, they have a negative effect on CO_2_ uptake and the energy investment capacity, and the RGR is affected [11,13,18,43]. The RGR is closely and positively associated with the SLA, as it is an indicator of the plant’s investment capacity [43,44]. PL (compared to PO × PL) exhibited lower values of both the RGR and SLA and thus, it had the shortest stem length. The SLA is highly plastic in several environmental gradients (light, nutrients, and water stress) and its responses are consistent in different groups of plants, including orchids [46,47]. It is related to the LWC, SC, RGR, and biochemical changes [41,42,43,44,45]. The unchanged SLA in PO × PL may be explained because this genotype exhibited higher LWC, SC, and RGR, as well as an accumulation of several osmolytes and salicylic acid (discussed later), allowing it to conserve water without having to reduce the leaf area [41,43].

### 3.2. Physio-Chemical Responses

**Figure 7:**
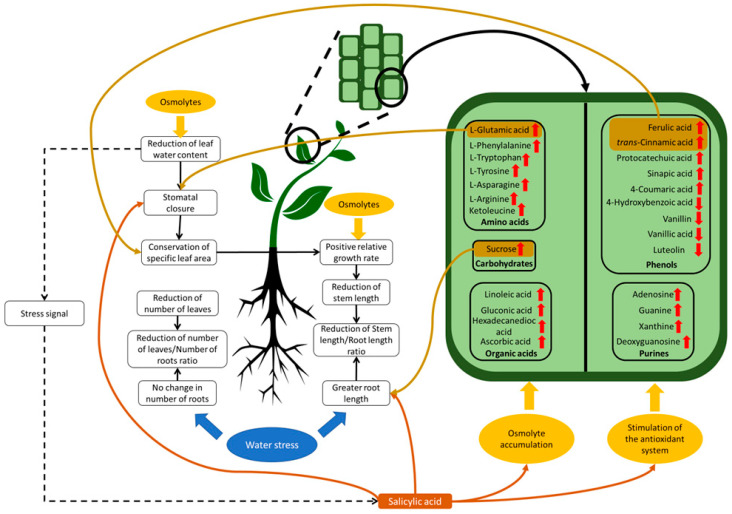
Morphological and physio-chemical responses of vanilla hybrids exposed to PEG-induced water stress. Water-stress-tolerant plants exhibit changes in morphology such as root elongation and reduction in leaf production and size, which increase water uptake. Additionally, stomatal closure is induced. Primary and secondary metabolism is modified in order to promote the accumulation of metabolites that act as osmolytes and antioxidants. Red arrows indicate an increase in the content of certain metabolites, while purple arrows indicate a decrease. Figure includes data from the present study and from the literature [11,14,16,17,18,19,20,40,41,45,46,47,48,49,57,58,59,60,61,62,73,74,75].

Under water stress, plants often modify primary and secondary metabolism with the aim of accumulating metabolites that act as osmolytes and antioxidants [16,45]. Changes in the metabolic response of both hybrids can be observed either in the heatmap or the PCA (Figure 4A,C). Both hybrids exhibited metabolic changes at 20 days of water stress exposure, as has been seen in other drought-tolerant plants [10,35]. In contrast, PL at 20 days of exposure did not change, suggesting that this species might be slower to modify its metabolism. This could be one of the reasons why PL was more susceptible to water stress [36]. Both hybrids accumulated carbohydrates at 20 and 40 days of water stress exposure (Tables S3–S6). Sucrose is reported to act as an osmolyte and antioxidant, and it could contribute to osmotic maintenance and ROS clearance in the system, and promote root elongation (Figure 7) [19,20,21]. Amino acids are an important part of osmotic regulation because they are both osmolytes and antioxidants [33,48]. In both hybrids (compared to PL) we found the accumulation of L-asparagine, ketoleucine, L-glutamic acid, L-arginine, L-tryptophan, L-tyrosine, and L-phenylalanine (Tables S3–S6), suggesting they play a role in the response to water stress conditions (Figure 7).

Regarding purines, xanthine was observed in PL × PO at 20 and 40 days of water stress exposure, and deoxyguanosine in PO × PL just at 20 days, while guanine and adenosine were present in both hybrids at both time points (Tables S3–S6). Purines are suggested to play a key role in tolerance to water stress given their antioxidant activity, as it has been reported in orchids of the genus *Dendrobium* [15,16]. In addition, they participated in other metabolic processes, such as the synthesis of nucleic acids, as precursors for the synthesis of primary and secondary metabolites and as a source of energy [14,15,16,48,49,50,51].

In vascular plants, organic acids contribute to osmotic maintenance, in addition to the cell structure, energy storage, and signaling, mitigating the negative impact of environmental stressors [15,33,52]. Linoleic acid, gluconic acid, and hexadecanedioic acid were accumulated at 20 and 40 days of water stress exposure in both hybrids, potentially because of their antioxidant activity [53,54,55] Ascorbic acid was also found (Tables S3–S6), which may have a role as growth promotor [56].

The change in the concentration of phenols in plants after water stress exposure has been previously reported [16,48]. Phenylalanine (precursor of phenolic compounds in the shikimate pathway), ferulic acid, *trans*-cinnamic acid, sinapic acid, 4-coumaric acid, and protocatechuic acid were found in higher concentrations in both hybrids compared to PL. This may reflect their osmoprotective and antioxidant activity, and because they might have contributed to maintain photosynthesis, as has been reported [10,57,58,59,60,61,62,63,64,65] (Table S7). The accumulation of these phenolic compounds could also be related to the greater RGR value found in both hybrids (Figure 7). On the other hand, 4-hydroxybenzoic acid, vanillic acid, vanillin, and luteolin decreased in all the plants studied when exposed to water stress (Table S7), which is possibly related to ROS clearance [66,67,68,69,70,71].

Salicylic acid only appeared in PO × PL. This phenolic compound is commonly called “the stress hormone” since it activates various plant defense mechanisms under biotic and abiotic stress [72]. The mechanisms of action of salicylic acid include the accumulation of osmolytes such as amino acids and soluble sugars in order to maintain osmotic homeostasis [73]; however, it also promotes the production of secondary/specialized metabolites, such as terpenes, other phenols, and nitrogenous compounds, stimulating the antioxidant system [72,73,74]. In addition to biochemical responses, salicylic acid can play a role in morphological and physiological responses, since it promotes root elongation, as well as stomatal closure [74,75] (Figure 7), which agrees with our results. The presence of salicylic acid only in PO × PL might be related to a better response to water stress, in comparison with the reciprocal hybrid and the parental genotype.

Overall, both hybrids had multiple responses to tolerate PEG-induced water stress. However, the PO × PL hybrid exhibited better tolerance than the reverse cross. In plant hybrids, there is usually a greater expression of the characteristics of the ovule donor over those of the pollen donor [76,77]. This may partly reflect the expression of maternal traits in chloroplasts and mitochondria DNA [78]. Both organelles are usually inherited from maternal parent [78,79]. Another plausible explanation for the discrepancy between the reciprocal hybrids is epigenetic regulation, since this regulation in plants is more sensitive to environmental stress [80,81]; however, the hypothesis must be tested in the future. Although *V. pompona* has been previously used in hybridization programs [76,82,83], it has rarely been used as a maternal species. The present study suggests that in future work, *V. pompona* should be used as a donor of ovules instead of pollen, and also, future work must provide specific evidence of the advantages of these *V. pompona* × *V. planifolia* hybrids over the parental species.

## 4. Materials and Methods

### 4.1. Biological Material

*In vitro* seedlings of *V. planifolia* (PL), *V. planifolia* × *V. pompona* (PL × PO) and *V. pompona* × *V. planifolia* (PO × PL) were used [84]; the first species mentioned is the ovule donor and the second is the pollen donor. Previous subcultures were performed in full-strength Murashige and Skoog medium (MS) supplemented with 6-benzylaminopurine at 0.2 mg/L [85] for vegetative multiplication to acquire enough plant material. Once the seedlings reached 2 cm in height and generated at least one root of 1 cm in length [86], they were subcultured in MS without PEG (control group, −0.08 mPa), and MS medium with PEG 5% *w*/*v* was added to induce moderate water stress (−0.49 mPa) [31]. The water potential in the medium was calculated using the following formula [87]:

φ = 0.84[PEG]^2^T − 118[PEG]^2^ − 0.267[PEG]T − 11.8[PEG]

where φ: water potential in megapascals (mPa); [PEG]: grams of PEG per milliliters of distilled water; and T: temperature of the medium (°C). There was one vanilla seedling per bottle, 10 repetitions per treatment, and they were placed in random blocks. The seedlings were placed in glass jars with a capacity of 235 mL (10.51 cm high × 6.5 cm diameter) on 25 mL of culture medium. Exposure to PEG-induced water stress lasted five weeks. The PEG-induced water stress condition was established at the beginning of the experiment and no further evaluations of the culture medium were conducted [33]. However, we performed comparisons at the same time between water-stressed versus full-hydrated (control groups) seedlings and between hybrids versus the parental genotype. In this work, *V. pompona* seedlings were not included because of the difficulty of germinating them in in vitro conditions.

### 4.2. Morphological and Physiological Responses to Water Stress

The increase in shoot length was recorded once a week during the five weeks. The comparison between treatments was made from the final values. The relative growth rate (RGR) was calculated from the weekly measurements, using the following formula [88]:

$$RGR=\frac{LnM_{2}-LnM_{1}}{\Delta t}$$

where *LnM*_1_: natural logarithm of the initial measurement and *LnM*_2_: natural logarithm of the following measurement. The length of the longest root produced by each plant was recorded at the end of the stress exposure time. The number of leaves and roots produced by the vanillas under the two water conditions and the leaf: root ratio and stem: longest root ratio were calculated. At the end of the period of exposure to water stress, three seedlings for each treatment were randomly selected, and one leaf of each seedling was harvested to be used for the evaluation of stomatal conductance (SC), specific leaf area (*SLA*), and leaf water content (LWC). Stomatal conductance was measured on the abaxial side of the leaves using a porometer (Decagon Devices, model SC-1); the vapor flow over the leaf surface was recorded (µ mol/m^2^ s) hourly between 9:00 and 14:00 h. For the determination of SLA, the area of the freshly cut leaves was measured with the *Imagej* 1.8.0 program [89] and was calculated with the SLA formula for in vitro seedlings [90]:

$$SLA=\frac{fresh\;leaf\;area\;\left({\mathrm{cm}}^{2}\right)}{dry\;leaf\;weight\;\left(\mathrm{mg}\right)}$$

For the measurement of the leaf water content, the leaves of each treatment were weighed at the moment of being cut (fresh weight), and later, they were dried in an oven at 60 °C for 24 h to obtain the dry weight [91]. The leaf water content was calculated as the difference between fresh weight and dry weight.

### 4.3. Physio-Chemical Response to Water Stress

#### 4.3.1. Untargeted Metabolomics

Seedling leaves of each treatment were harvested at 20 and 40 days of exposure to water stress. The leaves were placed in liquid nitrogen immediately after being cut and then stored at −80 °C until subsequent analyses. The samples were lyophilized (Freezone 1, Labconco, Kansas City, MO, USA), and pulverized in a mortar and pestle with liquid nitrogen. To obtain the crude extracts, accelerated solvent extraction system was used (ASE350, Dionex, Thermo Scientific, Waltham, MA, USA) [44]. The solvent used was methanol (HPLC grade) and water (MilliQ) in 80:20 ratio. Excess solvent was removed by evaporation under reduced pressure in rotary evaporator (Rotovap^®^ RII, Büchi, Newmarket, UK). From the resulting extracts, 500 µL of each sample was taken, and they were then placed in 1.5 mL UPLC tubes. The extract was filtered using a 0.45 µm filter prior to analysis. The samples were injected at a concentration of 20 mg/mL. The samples were evaluated in triplicates.

For the identification of distinctive metabolites associated with water stress, untargeted metabolomic analyses were carried out [92]. It was performed on an ultra-high-performance liquid chromatography system (UPLC, Acquity class I, Waters™, Milford, MA, USA), coupled to a high-resolution quadrupole time-of-flight mass spectrometer (QTOF, HDMI Synapt G2-Si model, Waters™). Chromatographic separation was carried out on an Acquity BEH column. (1.7 µm, 2.1 × 50 mm) at a temperature of 40 °C and 15 °C for the sample. The mobile phase consisted of (A) water and (B) acetonitrile, both with 0.1% formic acid. The conditions of the mobile phase gradient were 0–13 min linear gradient 1–80% B, 13–14 min 80% B isocratic, and 14–15 min linear gradient 80–1% B (total analysis time was 20 min). The flow rate was 0.3 mL/min and 5 µL of each extract was injected.

Mass spectrometry analysis was performed with an electrospray ionization source in positive and negative mode. The sampling cone, capillary, and source offset voltages were 3000, 40, and 80 V, respectively. The source temperature was 100 °C and the desolvation temperature was 20 °C. The desolvation gas flow was 600 L/h and the nebulizer pressure was 6.5 bar. The mass range was from 50 to 1200 Da, function 1 CE, and 6 V; function 2 CER 10–30 V and exploration time 0.5 s. Leucine–enkephalin (556.2771 [M + H]^+^; 554.2615 [M−H]^−^) was used as reference solution. Mass/charge ratios (*m*/*z*), retention time (RT), and peak intensity (total counts) were obtained and analyzed using Masslynx and MarkerLynx software (version 4.1, Waters™).

#### 4.3.2. Identification of Phenolics Compounds by UPLC-MS-MS

The extracts prepared for untargeted metabolomics were also used for the identification and quantification of individual phenolic compounds in leaves. Phenolic-targeted metabolomics was performed by ultra-high performance liquid chromatography (Agilent 1290 series) coupled with a triple quadrupole mass spectrometer (Agilent 6460), with a dynamic multiple reaction monitoring (dMRM) acquisition method, following the protocol established by [93] that included a total of 60 phenolic compounds. The chromatographic analysis was carried out on a ZORBAX SB-C18 column (1.8 μm, 2.1 × 50 mm; Agilent Technologies) with the column oven temperature at 40 °C. The mobile phase consisted of (A) water and (B) acetonitrile, both containing 0.1% formic acid. The gradient conditions of the mobile phase were as follows: 0 min 1% B, 0.1–40 min linear gradient 1–40% B, 40.1–42 min linear gradient 40–90% B, 42.1–44 min isocratic 90% B, 44.1–46 min linear gradient 90–1% B, and 46.1–47 min 1% B isocratic (total run time 47 min). The flow rate was 0.3 mL/min, and 2 μL was the sample injection volume. The ESI source operated in positive and negative ionization modes. The desolvation temperature was 300 °C, the cone gas (N_2_) flow was 5 L/min, the nebulizer pressure was 45 Psi, the sheath gas temperature was 250 °C, the sheath gas flow was 11 L/min, the capillary voltage (positive and negative) was 3500 V, and the nozzle voltage (positive and negative) was 500 V. The fragmentor voltage was 100 V and the cell accelerator voltage was 7 V for all compounds. The identity was confirmed by co-elution with authentic standards under the same analytical conditions described above for each compound. For quantitation of each phenolic compound a calibration curve in a concentration range of 1–9 μM was prepared (r^2^ values ≥ 0.97 were considered for the linearity range). The data were processed using the MassHunter Workstation Software, version B.06.00 (Agilent Technologies). The results were expressed as μg/g of sample (dry weight).

### 4.4. Statistical Analysis

For the morphological and physiological responses, two-level factorial arrangement was used: PEG concentration and vanilla genotype. Total length, longest root length, and the relationships between total length/root length and number of leaves/number of roots were analyzed using generalized linear model (GLM), gamma distribution, and inverse link function (*p* < 0.05). The number of leaves and roots produced was analyzed using GLM, Poisson distribution, logarithm link function (*p* < 0.05). RGR, SLA, stomatal conductance, and leaf water content, which were then analyzed by two-way analysis of variance (ANOVA) and Tukey’s post hoc test (*p* < 0.05). R 4.0.3 software was used [94], with packages Rmisc [95], agricolae [96], multcomp [97], and ggplot2 [98].

For the biochemical response, the intensities of the RT_m/z signals were used to elaborate heatmaps, PCA, network analysis, and volcano plots, using the MetaboAnalyst 5.0 software [99]. The main purpose of heatmap is to display grouping based on the abundance of the different spectrometric features (m/z_Rt) detected. The analysis of the enrichment of metabolic pathways was performed through the Mummichog algorithm, using the same MetaboAnalyst 5.0 software. For this, the KEGG database (Kyoto Encyclopedia of Genes and Genomes) was used [100], with *Oryza sativa* library for monocots. The RT-m/z signals that contributed to the discrimination between analyzed groups (fold change (FC) values greater or equal than 2) were considered differential chemical markers between conditions [101,102,103,104]. For the tentative identification of the distinctive metabolites (markers) that could be associated with changes in water potential, the KEGG databases were used [105] with a literature review, with a maximum mass error less than or equal to ±5 parts per million (ppm). The comparison between the concentration of the quantified phenols was carried out by two-way ANOVA and Tukey’s post hoc test (*p* < 0.05).

## 5. Conclusions

This is the first study in which both the morphological and physio-chemical responses to water stress of vanilla are addressed simultaneously. Additionally, it represents one of the few studies in which *V. planifolia* physiology is studied. Tolerance to PEG-induced water stress was achieved through root elongation to increase the water uptake and reduction in the number of leaves, as well as in the stomatal opening and the SLA, in order to reduce water loss. We observed an accumulation of carbohydrates, amino acids, purines, organic acids, and phenols; osmolyte and antioxidant activity was needed to achieve tolerance to water stress. The performance shown by the hybrid organisms suggests their ability to resist PEG-induced water stress under in vitro conditions. The *V. pompona* × *V. planifolia* hybrid exhibited the best PEG-induced water stress performance, possibly due to the maternal inherence of *V. pompona*. Vanilla hybrids are a potential alternative to the traditional cultivation of *V. planifolia*, especially given the difficulties that this crop will face due to the lack of water as a result of climate change; however, future studies should be carried out to determine the composition and content of the aromatic compounds in the fruits of these organisms. The approach of this study should be extended to other hybrids of *V. planifolia* and *V. pompona*, and also, to varieties of both species that are currently in cultivation, to determine which are likely to be best adapted for growth in water-limited conditions. These efforts will help protect *Vanilla* farmers from future extreme weather events and the *Vanilla* industry and consumers from future shortages and price fluctuations.

## Data Availability

Not applicable.

## Supplementary Materials

The following supporting information can be downloaded at: https://www.mdpi.com/article/10.3390/ijms24054690/s1.
