# Supplementary material for: Morphological and Physio-Chemical Responses to PEG-Induced Water Stress in Vanilla planifolia and V. pompona Hybrids
Source: Int J Mol Sci. 2023 Feb 28;24(5):4690. doi: 10.3390/ijms24054690 (PMC10003678; doi:10.3390/ijms24054690)
Supplement: Supplementary file 1 [file ijms-24-04690-s001.zip › ijms-2215395-supplementary.pdf]

Table S1. Metabolic pathways identified in *Vanilla planifolia* (PL), *V. planifolia* × *V. pompona* (PL × PO) and *V. pompona* × *V. planifolia* (PO × PL) hybrids when it was considered all signals detected. Determination by Mummichog algorithm, KEGG database, and *Oryza sativa* library.

Pathway	Total	Expected	Hits	p value	-LOG10(p)	Holm adjust	FDR	Impact
Aminoacyl-tRNA biosynthesis	46	1.0164	6	0.00035338	3.4518	0.033571	0.033571	0
Biosynthesis of unsaturated fatty acids	22	0.4861	4	0.0010822	2.9657	0.10173	0.051407	0
Sphingolipid metabolism	17	0.37562	3	0.0053893	2.2685	0.50121	0.15131	0.28846
Arginine biosynthesis	18	0.39772	3	0.0063709	2.1958	0.58612	0.15131	0.30972
Phenylalanine, tyrosine and tryptophan biosynthesis	22	0.4861	3	0.011324	1.946	1	0.17929	0.02152
Starch and sucrose metabolism	22	0.4861	3	0.011324	1.946	1	0.17929	0.0889
Arginine and proline metabolism	28	0.61867	3	0.022022	1.6571	1	0.29887	0.28609
Phenylpropanoid biosynthesis	35	0.77334	3	0.039642	1.4018	1	0.47075	0.03391
Butanoate metabolism	17	0.37562	2	0.052299	1.2815	1	0.55205	0
Porphyrin and chlorophyll metabolism	47	1.0385	3	0.082235	1.0849	1	0.67754	0.02435
Alanine, aspartate and glutamate metabolism	22	0.4861	2	0.082996	1.0809	1	0.67754	0.32374
Linoleic acid metabolism	4	0.088382	1	0.085584	1.0676	1	0.67754	1
Cyanoamino acid metabolism	26	0.57448	2	0.11063	0.95613	1	0.74619	0
Glutathione metabolism	27	0.59658	2	0.11788	0.92855	1	0.74619	0.05016
Galactose metabolism	27	0.59658	2	0.11788	0.92855	1	0.74619	0.11896
Isoquinoline alkaloid biosynthesis	6	0.13257	1	0.12567	0.90075	1	0.74619	0.41176
Tropane, piperidine and pyridine alkaloid biosynthesis	8	0.17676	1	0.16406	0.785	1	0.91681	0
Nitrogen metabolism	12	0.26515	1	0.236	0.62709	1	1	0
Phenylalanine metabolism	12	0.26515	1	0.236	0.62709	1	1	0.42308
Cutin, suberine and wax biosynthesis	14	0.30934	1	0.26969	0.56914	1	1	0
Tyrosine metabolism	18	0.39772	1	0.3328	0.47782	1	1	0.16757
Pentose phosphate pathway	19	0.41981	1	0.34773	0.45875	1	1	0
Valine, leucine and isoleucine biosynthesis	22	0.4861	1	0.39062	0.40825	1	1	0
Pantothenate and CoA biosynthesis	23	0.5082	1	0.4043	0.3933	1	1	0.14039
Tryptophan metabolism	23	0.5082	1	0.4043	0.3933	1	1	0.17241
Purine metabolism	63	1.392	2	0.40988	0.38734	1	1	0.00126

alpha-Linolenic acid metabolism	27	0.59658	1	0.4561	0.34094	1	1	0.11368
Glyoxylate and dicarboxylate metabolism	29	0.64077	1	0.48034	0.31845	1	1	0.0531
Glycine, serine and threonine metabolism	33	0.72915	1	0.52572	0.27924	1	1	0
Ubiquinone and other terpenoid-quinone biosynthesis	35	0.77334	1	0.54695	0.26205	1	1	0
Valine, leucine and isoleucine degradation	37	0.81753	1	0.56726	0.24622	1	1	0.00991
Cysteine and methionine metabolism	46	1.0164	1	0.64825	0.18826	1	1	0.02392
Flavonoid biosynthesis	47	1.0385	1	0.65628	0.18291	1	1	0.00032

FDR: value adjusted using False Discovery Rate

Table S2. Metabolites tentatively identified in *Vanilla planifolia* (PL), *V. planifolia* × *V. pompona* (PL × PO) and *V. pompona* × *V. planifolia* (PO × PL) hybrids when it was considered all signals detected.

RT	m/z	Adduct	Tentative metabolite	KEGG ID	Mass difference	Fragments
0.39	131.082	$[M-H]^-$	Ornithine	C00077	0.0006	
0.4	175.1192	$[M+H]^+$	L-Arginine	C00062	0.0002	158.0927, 116.0709
0.43	84.04453	$[M-H_2O+H]^+$	1-Aminocyclopropanecarboxylic acid	C01234	0.0002	
0.44	287.0557	$[M-H]^-$	Fustin	C01378	0.0004	109.0402
0.45	517.1402	$[M-H_2O-H]^-$	Galabiose	C00760	0.0003	
0.45	133.0611	$[M+H]^+$	L-Asparagine	C00152	0.0003	
0.46	195.0506	$[M-H]^-$	Gluconic acid	C00257	0.0004	
0.47	539.1372	$[M+Cl]^-$	Disaccharide	C00492	0.0007	
0.48	377.0844	$[M+Cl]^-$	Sucrose	C00089	0.0007	341.1088, 161.0447, 85.0286, 71.0135
0.5	503.1603	$[M-H]^-$	Dextrin	C00721	0.0015	
0.64	262.1286	$[M+H]^+$	Lotaustralin	C08334	0.0001	
0.77	130.0499	$[M-H_2O+H]^+$	L-Glutamic acid	C00025	0.0001	
0.87	180.0658	$[M-H]^-$	L-Tyrosine	C00082	0.0008	
1.13	268.1039	$[M+H]^+$	Adenosine	C00212	0.0001	136.062
1.2	152.0569	$[M+H]^+$	Guanine	C00242	0.0002	
1.58	164.0711	$[M-H]^-$	L-Phenylalanine	C00079	0.0006	
2.22	203.082	$[M-H]^-$	L-Tryptophan	C00078	0.0006	
5.19	187.0022	$[M+Cl]^-$	Xanthine	C00385	0.0001	
6.2	177.0551	$[M-H_2O+H]^+$	trans-Ferulic acid	C01494	0.0005	
9.31	245.0959	$[M+H]^+$	Biotin	C00120	0.0001	
10.26	89.0599	$[M+H]^+$	Butyric acid	C00246	0.0002	
10.27	131.0707	$[M+H]^+$	Ketoleucine	C00233	0.0004	
11.25	302.3053	$[M+H]^+$	Sphinganine	C00836	0.0001	
11.46	318.2989	$[M+H]^+$	Phytosphingosine	C12144	0.0014	
13.22	279.2319	$[M+H]^+$	α-Linolenic acid	C06427	0.0000	
13.6	341.0936	$[M-H_2O+H]^+$	Pantetheine 4'-phosphate	C01134	0.0006	
13.98	137.0238	$[M+Cl]^-$	*p*-Hydroxybenzoic acid	C00156	0.0001	
14.34	211.0008	$[M+Cl]^-$	Ascorbic acid	C00072	0.0001	
14.41	300.0811	$[M+Cl]^-$	Thiamine	C00378	0.0001	

16.39	279.2322	$[M+H]^+$	γ-Linolenic acid	C06426	0.0003
16.95	609.2705	$[M-H_2O+H]^+$	Red chlorophyll catabolite	C18022	0.0002
17.16	282.2792	$[M-H_2O+H]^+]$	Sphingosine	C00319	0.0001
17.39	337.2346	$[M+Na]^+$	9,10-Epoxystearic acid	C19620	0.0003
17.51	609.2695	$[M-H_2O+H]^+$	Red chlorophyll catabolite	C18022	0.0012
17.95	593.275	$[M+H]^+$	Pheophorbide A	C18021	0.0008
18.81	284.2941	$[M-H_2O+H]^+$	Sphinganine	C00836	0.0007

RT: Retention time (minutes), m/z: observed mass/charge ratio. The mass difference is expressed in Daltons.

Table S3. Differential metabolites (fold change > 2) tentatively identified in *Vanilla planifolia* × *V. pompona* (PL × PO) respect to *V. planifolia* (PL) due to exposure to 20 days of water stress.

RT	m/z	FC	p value	Compound name	Ionization mode
10.27	131.0707	9060.9	0.024603	Ketoleucine	Positive
0.46	195.0506	126.06	0.035725	Gluconic acid	Negative
0.87	180.0658	75.387	0.029764	L-Tyrosine	Negative
10.26	89.0599	50.396	0.00028234	Butyric acid	Positive
1.58	164.0711	40.309	0.0011963	L-Phenylalanine	Negative
0.45	133.0611	23.918	0.000003	L-Asparagine	Positive
0.47	539.1372	20.907	0.01575	Disaccharide	Negative
1.2	152.0569	20.1576	0.000007	Guanine	Positive
0.48	377.0844	12.03	0.014075	Sucrose	Negative
13.98	137.0238	6.7492	0.0090387	*p*-Hydroxybenzoic acid	Negative
0.45	517.1402	5.7962	0.003182	Galabiose	Negative
0.77	130.0499	5.6323	0.0031837	L-Glutamic acid	Negative
2.22	203.082	5.4241	0.000006	L-Tryptophan	Negative
1.13	268.1039	3.3031	0.0065064	Adenosine	Positive
17.16	282.2792	2.9707	0.0027435	Sphingosine	Positive
5.19	187.0022	2.9439	0.0032244	Xanthine	Negative
15.23	309.2027	2.9392	0.005795	Hexadecanedioic acid	Positive
9.31	245.0959	2.9177	0.003479	Biotin	Positive
17.39	337.2346	2.7698	0.0017257	9,10-Epoxystearic acid	Positive
18.29	593.2746	2.7353	0.0033474	Pheophorbide A	Positive
18.81	284.2941	2.4795	0.011258	Sphinganine	Positive

RT: Retention time (minutes), m/z: observed mass/charge ratio. FC: Fold Change.

Table S4. Differential metabolites (Fold change > 2) tentatively identified in *Vanilla planifolia* × *V. pompona* (PL × PO) respect to *V. planifolia* (PL) due to exposure to 40 days of water stress.

RT	m/z	FC	p value	Compound name	Ionization mode
0.48	377.0844	511.49	0.01856	Sucrose	Negative
10.27	131.0707	77	0.027956	Ketoleucine	Positive
0.45	517.1402	47.987	0.0021397	Galabiose	Negative
1.2	152.0569	20.1576	0.000007	Guanine	Positive
10.26	89.0599	17.191	0.0060712	Butyric acid	Positive
16.39	279.2322	8.1653	0.00061716	γ-Linolenic acid	Positive
13.22	279.2319	5.8874	0.000005	α-Linolenic acid	Positive
19.65	307.2628	5.6019	0.000001	8,11,14-Eicosatrienoic acid	Positive
1.58	164.0711	5.5878	0.000009	L-Phenylalanine	Negative
2.22	203.082	5.4241	0.000006	L-Tryptophan	Negative
14.34	211.0008	4.2098	0.0071595	Ascorbic acid	Negative
5.19	187.0022	3.9439	0.0032244	Xanthine	Negative
1.13	268.1039	3.3031	0.0065064	Adenosine	Positive
0.47	539.1372	2.9092	0.0071325	Disaccharide	Negative
0.46	195.0506	2.179	0.089374	Gluconic acid	Negative

RT: Retention time (minutes), m/z: observed mass/charge ratio. FC: Fold Change.

Table S5. Differential metabolites (Fold change > 2) tentatively identified in *Vanilla pompona* × *V. planifolia* (PO × PL) respect to *V. planifolia* (PL) due to exposure to 20 days of water stress.

RT	m/z	FC	p value	Compound name	Ionization mode
10.27	596.364	7491.6	0.0246	Ketoleucine	Positive
0.46	195.0506	135.9	0.032891	Gluconic acid	Negative
0.87	180.0658	81.445	0.026631	L-Tyrosine	Negative
10.26	89.0599	53.756	0.00046958	Butyric acid	Positive
1.58	164.0711	40.309	0.0011963	L-Phenylalanine	Negative
0.48	377.0844	27.818	0.038817	Sucrose	Negative
19.65	307.2628	23.108	0.001451	8,11,14-Eicosatrienoic acid	Positive
0.47	539.1372	22.604	0.01231	Disaccharide	Negative
0.45	133.0611	20.697	0.0000012801	L-Asparagine	Positive
1.2	152.0569	20.1576	0.0000723	Guanine	Positive
9.31	245.0787	16.16	0.00016983	Biotin	Positive
13.98	137.0238	7.2879	0.004255	*p*-Hydroxybenzoic acid	Negative
14.34	211.0008	6.6588	0.00051359	Ascorbic acid	Negative
0.4	175.1192	6.2712	0.0016807	L-Arginine	Negative
0.77	130.0499	6.1286	0.00066024	L-Glutamic acid	Negative
2.22	203.082	5.4241	0.000006	L-Tryptophan	Negative
13.22	279.2319	3.5684	0.0060272	α-Linolenic acid	Positive
1.13	268.1039	3.3031	0.0065064	Adenosine	Positive
18.81	284.2941	2.7612	0.011819	Sphinganine	Positive
17.39	337.2346	2.7327	0.0064241	9,10-Epoxystearic acid	Positive
15.23	309.2027	2.6302	0.020651	Hexadecanedioic acid	Positive

RT: Retention time (minutes), m/z: observed mass/charge ratio. FC: Fold Change.

Table S6. Differential metabolites (Fold change > 2) tentatively identified in *Vanilla pompona* × *V. planifolia* (PO × PL) respect to *V. planifolia* (PL) due to exposure to 40 days of water stress.

RT	m/z	FC	p value	Compound name	Ionization mode
0.45	133.0611	69.638	0.042323	L-Asparagine	Positive
10.27	596.364	46.451	0.044902	Ketoleucine	Positive
0.4	175.1192	22.534	0.0993	L-Arginine	Negative
1.2	152.0569	20.1576	0.0000723	Guanine	Positive
14.76	248.0784	20.072	0.044928	Deoxyguanosine	Negative
10.26	89.0599	15.358	0.03919	Butyric acid	Positive
0.77	130.0499	10.697	0.044146	L-Glutamic acid	Negative
14.34	211.0008	6.9971	0.049997	Ascorbic acid	Negative
0.48	143.0343	5.3417	0.043201	Sucrose	Negative
16.39	279.2322	4.9399	0.0066307	γ-Linolenic acid	Positive
0.45	517.1402	4.411	0.050389	Galabiose	Negative
13.98	137.0238	4.1834	0.050665	*p*-Hydroxybenzoic acid	Negative
13.22	279.2319	3.5684	0.0060272	α-Linolenic acid	Positive
18.81	284.2941	3.4985	0.0059782	Sphinganine	Positive
0.47	539.1372	3.4713	0.070816	Disaccharide	Negative
19.65	307.2628	3.3941	0.0064076	8,11,14-Eicosatrienoic acid	Positive
1.58	164.0711	3.3852	0.006183	L-Phenylalanine	Negative
1.13	268.1039	3.3031	0.0065064	Adenosine	Positive
2.22	203.082	3.2871	0.0066341	L-Tryptophan	Negative
17.39	337.2346	3.2855	0.0066476	9,10-Epoxystearic acid	Positive
0.87	180.0658	3.2844	0.071636	L-Tyrosine	Negative
11.46	318.2989	3.2716	0.0066876	Phytosphingosine	Positive
17.16	282.2792	3.2424	0.0069119	Sphingosine	Positive
0.64	262.1286	3.2254	0.046892	Lotaustralin	Positive
17.95	593.275	3.21	0.0071724	Pheophorbide A	Positive
0.46	195.0506	2.6691	0.083823	Gluconic acid	Negative

RT: Retention time (minutes), m/z: observed mass/charge ratio. FC: Fold Change.

Table S7. Identification and quantification of phenolic compounds in *Vanilla planifolia* × *V. pompona* (PL × PO) *V. pompona* × *V. planifolia* (PO × PL) and *V. planifolia* (PL) due to exposure to 20 and 40 days of water stress.

SAMPLES	Phenylalanine	Salicylic acid	Vanillic acid	Vanillin	Ferulic acid	Sinapic acid
PL-0 PEG-20	467.49 ± 3.96**b**	---	3.52 ± 0.08**b**	2.3 ± 0.04**c**	28.75 ± 0.62**a**	6.63 ± 0.04**b**
PL × PO-0 PEG-20	307.26 ± 2.14**e**	---	4.12 ± 0.1**a**	1.94 ± 0.04**d**	21.95 ± 0.03**d**	1.33 ± 0.04**g**
PO × PL-0 PEG-20	216.92 ± 1.64**h**	1.39 ± 0.03**a**	2.3 ± 0.01**c**	3.11 ± 0.08**b**	7.88 ± 0.09**h**	0.31 ± 0.02***ij**
PL-5 PEG-20	201.13 ± 1.01**i**	---	1.76 ± 0.05**e**	1.54 ± 0.04**e**	27.56 ± 0.27**b**	8 ± 0.03**a**
PL × PO-5 PEG-20	280.86 ± 1.39**f**	---	1.53 ± 0.02**f**	2.42 ± 0.05**c**	19.44 ± 0.31**e**	3.94 ± 0.15**d**
PO × PL-5 PEG-20	181.46 ± 2.3**j**	0.41 ± 0.03***b**	0.67 ± 0.02***i**	2.42 ± 0.07**c**	6.45 ± 0.06**i**	0.5 ± 0.02***hi**
PL-0 PEG-40	535.28 ± 2.06**a**	---	2.45 ± 0.1**c**	1.89 ± 0.03**d**	24.28 ± 0.1**c**	6.08 ± 0.04**c**
PL × PO-0 PEG-40	424.31 ± 2.99**c**	---	0.75 ± 0***hi**	1.96 ± 0.01**d**	7.77 ± 0.15**h**	2.88 ± 0.07**f**
PO × PL-0 PEG-40	272.01 ± 1.01**g**	---	2.04 ± 0.02**d**	4.34 ± 0.06**a**	7.43 ± 0.07**h**	0.21 ± 0.06***j**
PL-5 PEG-40	308.58 ± 1.57**e**	---	1.23 ± 0.05***g**	1.49 ± 0.04**e**	13.77 ± 0.1**g**	4.06 ± 0.11**d**
PL × PO-5 PEG-40	349.84 ± 2.59**d**	---	0.89 ± 0.05***h**	1.89 ± 0.05**d**	15.85 ± 0.21**f**	3.37 ± 0.03**e**
PO × PL-5 PEG-40	536.75 ± 6.59**a**	---	0.46 ± 0.02***j**	1.45 ± 0.07**e**	5.07 ± 0.13**j**	0.59 ± 0.01***h**

PL: *Vanilla planifolia*, PL × PO: *V. planifolia* × *V. pompona*, PO × PL: *V. pompona* × *V. planifolia*, 0 PEG: hydration status (control group, -0.08 mPa), 5 PEG: hydric stress (-0.49 mPa), 20: 20 days of water stress exposure, 40: 40 days of water stress exposure. Concentration is expressed in µg/g of dried extract, and it is shown the average of three determinations plus and minus the standard deviation. "---": Not identified. *: Value determined below the limit of quantification. Values with different letters are significantly different ($p < 0.05$), according to ANOVA and *post hoc* Tukey test.

Table S7 (continuation). Identification and quantification of phenolic compounds in *Vanilla planifolia* × *V. pompona* and *V. pompona* × *V. planifolia* hybrids, and the species *V. planifolia* due to exposure to 20 and 40 days of water stress.

**SAMPLES**	**4-Coumaric acid**	***trans*-Cinnamic acid**	**Luteolin**	**Protocatechuic acid**	**4-Hydroxybenzoic acid**
PL-0 PEG-20	42.3 ± 0.24**b**	0.83 ± 0.02**c**	---	0.11 ± 0.01*__d__	2.36 ± 0.05**a**
PL × PO-0 PEG-20	7.12 ± 0.02**k**	0.2 ± 0.03*__fg__	0.07 ± 0.01*__e__	0.4 ± 0*__c__	1.91 ± 0.02**b**
PO × PL-0 PEG-20	9.21 ± 0.05**j**	1.39 ± 0.03**b**	4.64 ± 0.05**a**	0.84 ± 0.01*__a__	1.5 ± 0.02**d**
PL-5 PEG-20	37.66 ± 0.25**d**	0.15 ± 0.01*__g__	---	---	1.45 ± 0.03**d**
PL × PO-5 PEG-20	38.56 ± 0.29**c**	0.17 ± 0.01**g**	---	0.04 ± 0*__f__	1.34 ± 0.02**e**
PO × PL-5 PEG-20	30.29 ± 0.49**f**	0.4 ± 0.01*__e__	---	0.09 ± 0.01*__e__	1.07 ± 0.02**g**
PL-0 PEG-40	35.24 ± 0.23**e**	0.25 ± 0.03*__f__	0.81 ± 0.07*__c__	---	1.29 ± 0.04**e**
PL × PO-0 PEG-40	28.12 ± 0.08**g**	0.87 ± 0.03*__c__	0.38 ± 0.02*__d__	---	0.84 ± 0.01*__h__
PO × PL-0 PEG-40	9.11 ± 0.07**j**	1.4 ± 0.05*__b__	4.71 ± 0.09**a**	0.8 ± 0.01*__b__	1.8 ± 0**c**
PL-5 PEG-40	22.38 ± 0.14**i**	0.26 ± 0.01*__f__	---	---	1.33 ± 0.03**e**
PL × PO-5 PEG-40	58.69 ± 0.61**a**	1.79 ± 0.02**a**	4.26 ± 0.06**b**	---	1.3 ± 0.03**e**
PO × PL-5 PEG-40	25.79 ± 0.31**h**	0.52 ± 0*__d__	---	0.09 ± 0*__e__	1.2 ± 0.02**f**

PL: *Vanilla planifolia*, PL × PO: *V. planifolia* × *V. pompona*, PO × PL: *V. pompona* × *V. planifolia*, 0 PEG: hydration status (control group, -0.08 mPa), 5 PEG: hydric stress (-0.49 mPa), 20: 20 days of water stress exposure, 40: 40 days of water stress exposure.Concentration is expressed in μg/g of dried extract, and it is shown the average of three determinations plus and minus the standard deviation. "---": Not identified. *: Value determined below the limit of quantification. Values with different letters are significantly different ($p < 0.05$), according to ANOVA and *post hoc* Tukey test.
